# Complete Genome Sequence of a Panton-Valentine Leukocidin-Negative Staphylococcus aureus Strain Isolated from a Patient with Pervasive Necrotizing Soft Tissue Infection

**DOI:** 10.1128/MRA.00295-20

**Published:** 2020-06-04

**Authors:** Yoshifumi Aiba, Shinya Watanabe, Rieko Tsukahara, Naoka Umemoto, Kanate Thitiananpakorn, Tanit Boonsiri, Feng-Yu Li, Kotaro Kiga, Yusuke Sato’o, Xin-Ee Tan, Yusuke Taki, Aa Haeruman Azam, Yuancheng Zhang, Teppei Sasahara, Toshio Demitsu, Longzhu Cui

**Affiliations:** a Division of Bacteriology, Department of Infection and Immunity, School of Medicine, Jichi Medical University, Shimotsuke, Japan; b Department of Dermatology, Jichi Medical University Saitama Medical Center, Saitama, Japan; University of Maryland School of Medicine

## Abstract

The association of Panton-Valentine leukocidin (PVL) toxin with necrotizing soft tissue infection (NSTI) caused by Staphylococcus aureus remains controversial. Here, we report the complete genome sequence of the PVL-negative S. aureus strain JMUB1273, isolated from a patient with pervasive NSTI.

## ANNOUNCEMENT

Necrotizing soft tissue infection (NSTI) is a life-threatening infection with a substantially high mortality rate (11% to 36%) and is occasionally caused by Staphylococcus aureus (1, 2). There are many staphylococcal toxins or extracellular products that are associated with the pathogenesis of NSTIs, including Panton-Valentine leukocidin (PVL) toxin, toxic shock syndrome toxin 1 (TSST-1), enterotoxin (SE) or enterotoxin-like (SEI) toxin, exfoliative toxin (ET), and phenol-soluble modulin (PSM). However, their individual involvement in the pathogenesis of NSTIs remains controversial and yet to be fully understood.

S. aureus strain JMUB1273 was isolated from a 61-year-old man with an NSTI on his upper back that occurred during dietary modification under glycemic control. Upon physical examination, a huge black lesion covering over half of the upper back beyond what appeared to be the confines of infection was observed, and pus was oozing from the area of the lesion. The patient’s laboratory risk indicator for necrotizing fasciitis (LRINEC) score was 8. Eventually, the patient was successfully treated with surgical drainage, negative pressure wound therapy, antibiotics, and glycemic control, followed by skin grafting. The bacterial strain was isolated from surgical wound drainage by growing it on a sheep blood agar plate (Nissui, Tokyo, Japan) under 5% CO_2_ at 35°C for 48 h. The strain was identified as S. aureus and oxacillin susceptible by the RAISUS S4 (Nissui, Tokyo, Japan) system for rapid bacterial identification and antimicrobial susceptibility testing.

Genomic DNA extraction and whole-genome sequence analysis were performed as described previously (3). Briefly, JMUB1273 genomic DNA was extracted from an overnight culture in tryptic soy broth (BD, NJ, USA) at 37°C using the NucleoBond AXG kit (TaKaRa Bio, Inc., Japan). A genomic library was prepared using a rapid barcoding sequencing kit (SQK-RBK004) with an input DNA amount of 400 ng according to the manufacturer’s protocol. To determine the whole-genome sequence, a MinION Mk-1B device (Oxford Nanopore Technologies [ONT], Oxford, United Kingdom) integrated with a FLO-MIN106 (R9.4.1) flow cell (ONT) was used, and a total of 126,370 reads (average size, 5,004 bp) was obtained using MinKNOW software (version 1.14.1; ONT). The ONT sequences were then demultiplexed and the potential remaining adaptors were trimmed using Porechop tools (version 0.2.4; https://github.com/rrwick/porechop); they were *de novo* assembled into a single contig by using Canu (version 1.8; http://canu.readthedocs.org/). To correct errors in the assembled sequences, 986,906 short reads were generated using the Nextera mate pair preparation kit (Illumina, Inc.) for library construction and the MiSeq reagent kit version 3 (Illumina, Inc.) for sequencing (4, 5). The short reads generated with the MiSeq platform (2 × 301-bp, paired-end format) were trimmed with the FASTQ toolkit version 2.0.0 (Illumina, Inc.) with a quality level of 30. Using the MiSeq short reads, the assembled sequences generated from the ONT reads were polished with Pilon (version 1.22; https://github.com/broadinstitute/pilon/) (6) and the CLC Genomics Workbench version 9.5.3 (Qiagen, Hilden, Germany). Gene annotation was performed with the Microbial Genome Annotation Pipeline or Prokka (version 1.13.36) (7). Default parameters were used for all software unless otherwise noted.

The whole-genome sequence determination and annotation showed that JMUB1273 harbors a single circular chromosome that is 2,733,771 bp long (coverage depth, 61.7×) with a GC content of 32.8% and encodes 2,640 predicted proteins, 59 tRNAs, and 6 rRNAs. Following a detailed analysis using multilocus sequence typing (MLST 2.0), we found that JMUB1273 belonged to sequence type 188 (ST188) and clonal complex CC1 (8, 9). Many staphylococcal toxins known to be associated with NSTIs, including PVL, TSST-1, SE, and ET, were not found in this genome. However, other potentially associated genes (1, 10) encoding virulence factors, such as gamma-hemolysin, leukocidin, aureolysin, serine proteases, staphylokinase, putative SEI protein, PSMβ1, and staphylococcal complement inhibitor, were identified in this genome.

### Data availability.

The genome sequence was deposited in DDBJ/GenBank under the accession number AP018922. The associated BioProject and BioSample accession numbers are PRJDB7266 and SAMD00134450, respectively. The raw data reads have been deposited in the DDBJ/Sequence Read Archive under the accession numbers DRA009801 (ONT) and DRA009505 (Illumina).
